# Assembling the genome of the African wild rice *Oryza longistaminata* by exploiting synteny in closely related *Oryza* species

**DOI:** 10.1038/s42003-018-0171-y

**Published:** 2018-10-05

**Authors:** Stefan Reuscher, Tomoyuki Furuta, Kanako Bessho-Uehara, Michele Cosi, Kshirod K. Jena, Atsushi Toyoda, Asao Fujiyama, Nori Kurata, Motoyuki Ashikari

**Affiliations:** 1 Bioscience and Biotechnology Center, Nagoya University, Furo-cho, Chikusa-ku, Nagoya, Aichi 464-8601 Japan; 2 International Rice Research Institute, DAPO Box 7777, Metro Manila, 1301 Philippines; 3 Center for Information Biology, National Institute of Genetics, Mishima, 411-8540 Japan; 4 Genetic Strains Research Center, National Institute of Genetics, Mishima, 411-8540 Japan

## Abstract

The African wild rice species *Oryza longistaminata* has several beneficial traits compared to cultivated rice species, such as resistance to biotic stresses, clonal propagation via rhizomes, and increased biomass production. To facilitate breeding efforts and functional genomics studies, we de-novo assembled a high-quality, haploid-phased genome. Here, we present our assembly, with a total length of 351 Mb, of which 92.2% was anchored onto 12 chromosomes. We detected 34,389 genes and 38.1% of the genome consisted of repetitive content. We validated our assembly by a comparative linkage analysis and by examining well-characterized gene families. This genome assembly will be a useful resource to exploit beneficial alleles found in *O. longistaminata*. Our results also show that it is possible to generate a high-quality, functionally complete rice genome assembly from moderate SMRT read coverage by exploiting synteny in a closely related *Oryza* species.

## Abstract (web summary)

Stefan Reuscher et al. assembled the genome of an African wild rice species to facilitate breeding efforts and functional genomic studies. They used SMRT sequencing, chromosomal synteny between rice species, and a linkage map to assemble the 351 Mb genome into 12 chromosomes.

## Introduction

The *Oryza* genus in the grass family (*Poaceae*) contains the cultivated rice species *Oryza sativa* from Asia and *Oryza glabberima* from Africa. In addition, the *Oryza* genus consists of at least 20 wild species with a global distribution which contain an enormous reservoir of untapped variation^1,2^. The genus contains mostly diploid (2*n* = 24) species with occasional tetraploids (4*n* = 48) and can be divided into genome types based on their ability for interbreeding. Genome types range from diploid AA to tetraploid KKLL, with both commercially used species (*O*. *sativa* and *O. glabberima*) belonging to the AA type. Recent advances in DNA sequencing technology have enabled the (re-)sequencing of several commercial and wild AA genome-rice varieties, although some reported assemblies do not attempt to reconstruct full chromosomes^3–10^.

The wild rice *Oryza longistaminata* is of the AA genome type. It is found in tropical regions of western Africa near fresh water and in swampy areas^1^. It is rarely used for human consumption, but holds a number of beneficial traits, such as resistance to bacterial blight linked to the *Xa21* locus^11^, perennial growth, and a high biomass production. The latter two are likely associated with the ability of *O. longistaminata* to propagate clonally via rhizomes. Efforts have been made to transfer beneficial alleles from *O. longistaminata* into commercial varieties by evaluating the agricultural potential of introgressed chromosomal segments from *O. longistaminata* into a commercial background^12,13^. In addition to breeding efforts, *O. longistaminata* is also used to study the genetic basis and the development of rhizomes^5,14–16^.

The assembly of a complete plant genome provides a strong basis for functional genomics studies or for efforts to identify candidate genes through traditional mapping approaches. However, truly chromosome-complete plant genomes are still not a trivial achievement^17^. Among the cereals, the rice genome is more amendable to assembly due to the fact that it is less repetitive and its size is generally less than 500 Mb. For large cereal genomes such as barley (ca. 5 Gb), a full chromosome assembly was achieved by a combination of ultra-high coverage of small reads, BAC libraries, manual curation, and a number of technologies that produce long-range positional information, such as optical mapping or chromosome conformation capture^18^. For rice genome assembly, the same technologies can be used, however due to the smaller genome size, sequencing efforts should require less extensive resources.

The possibility to produce long reads (10–40 kb) from genomic DNA by single molecule real-time (SMRT) sequencing technology has enabled complete genome assemblies for diverse organisms, including notoriously repetitive plant genomes^19,20^. Despite the advantages of long-read sequencing, recent rice genomes assemblies still rely on supplemental technologies to provide large-scale genomic context of contig sequences^3,4^. In addition, short reads are still necessary to correct single nucleotide and small indel errors in the SMRT reads.

In this work, we assembled the genome of *O. longistaminata*, including 12 chromosome-scale sequences with alternative parental haplotypes. We used a comparatively moderate coverage (66×) of SMRT reads and exploited gene-synteny in the *Oryza* genus and a previously generated genetic map for our assembly. In total, we assembled 351 Mb of which 92.2% could be placed on 12 chromosomes. We furthermore validate our genome assembly for its usefulness in possible functional genomics studies and breeding efforts.

## Results

### Genome assembly and annotation

We sequenced DNA extracted from young leaves of one individual plant of *O. longistaminata* accession IRGC110404. In total, we used 16 PacBio SMRT V3 cells generating 22.6 Gb on 2.4 million reads (average read length: 9.3 kb) (Supplementary Figure 1). The total nuclear genome size of *O. longistaminata* was estimated to be around 340 Mb^5^, setting our average coverage to approximately 66-fold.

**Fig. 1:**
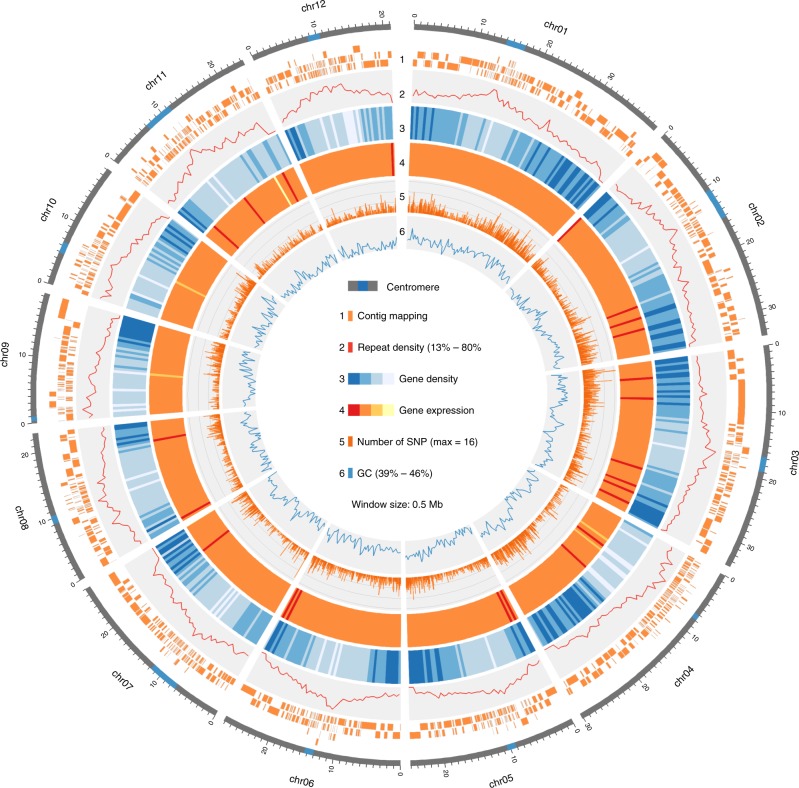
Circular overview of the 12 assembled *O. longistaminata* chromosomes. Circular overview plot showing basic features across the *O. longistaminata* genome assembly. From outermost to innermost ring: contig coverage of the pseudo-chromosome, density of repeat elements in % of total bp, density of genes in % of total bp, mean gene expression in RPKM in eight tissues, number of SNPs between *O. longistaminata* and *Oryza sativa* ssp. *japonica* cv. Nipponbare, % of GC. All tracks except contig mapping show binned data with a window size of 0.5 Mb. Axis limits are shown in the center of the plot. Data sources are fully described in the main text

Assembly using FALCON-UNZIP^21^ resulted in 1632 primary contigs with a total length of 350.56 Mb, an N50 of 554 kb and a maximum contig size of 7.29 Mb (Table 1). In addition, FALCON-UNZIP assembled 4229 contigs representing the alternative haplotype on the sister chromatid (haplotigs). Those alternative contigs had a total combined length of 258.67 Mb and an N50 of 148 kb (73.79% of the primary assembly). We also tried the Canu assembler^22^ with our raw data but found that FALCON-UNZIP performed better, possibly because of the heterozygosity found in the *O. longistaminata* genome (Supplementary Note 1 and Supplementary Table 1). For error-correction of the primary contigs, we first re-aligned the SMRT reads to the assembled contigs with blasr and then used quiver to correct 1.3 million insertions, 0.2 million deletions, and 0.61 million substitutions. In the next step, 18.35 million pairs of short reads (150 bp read length, 432 bp median insert size) were aligned to the contigs and an additional 0.24 million insertions and 38.7 thousand deletions were corrected. The polished contigs were arranged and oriented using a genetic map^23^ and exploiting gene synteny with *O. sativa* ssp. *japonica* (Supplementary Figure 2), resulting in the assembly of 12 pseudo-chromosomes with a total length of 323.95 Mb (92.2% placement rate) (Fig. 1). The completeness of the genome assembly was assessed by detecting a set of unique single copy genes in the genome assembly^24^. Out of 1440 unique single copy genes, 1360 (94.5%) were detected in our assembly. Gene models in the newly assembled genome were determined using a combination of computational gene prediction and expressed transcripts based on RNAseq data from eight diverse tissues. The final gene model set consisted of 34,389 genes with a median gene length of 2,700 bp. Using the Mercator annotation pipeline, MAPMAN bins (other than unassigned) could be detected for 20,121 genes^25^. Putative centromeric regions were identified on all chromosomes. Although we failed to identify telomeric repeat regions ([TTTAGGG]_*n*_) in our final assembly, such repeat structures were represented in the pool of error-corrected reads. The contig coverage of the final pseudomolecules tended to be less fragmented in the comparably gene-rich chromosome arms, while the centromeric regions were mostly reconstructed from shorter contigs. This might lead to inaccuracies in the assembly of such regions.

**Table 1:** Basic genome-wide statistics of the *O. longistaminata* assembly

Genome statistic	
Total size of assembled contigs (bp)	350,562,179
Total size of contigs anchored on chromosomes (bp)	324,081,576
Contig placement rate (%)	92.45
Number of contigs	1632
Longest contig (bp)	7,290,908
Contig N50 (bp)	553,927
Number of gene models	34,389
Median gene length (bp)	2700
Genic content (%)	40.19
Repeat content (%)	38.10
GC content (%)	42.71

### Whole genome alignment

**Fig. 2:**
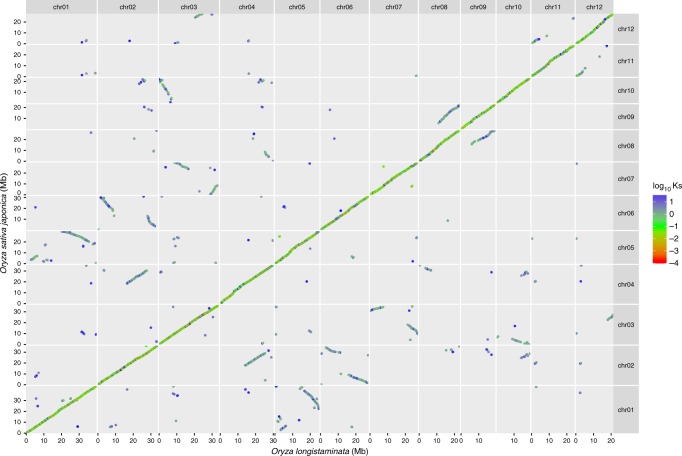
Genome-wide alignment of CDS from *O. longistaminata* and *O. sativa*. Pairwise comparisons of CDS from the *O. longistaminata* genome assembly and the *Oryza sativa* ssp. *japonica* cv. Nipponbare genome were used to detect syntenic chains of genes. Pairs of CDS are plotted according to their position in the *O. longistaminata* genome (*x*-axis) and *O. sativa* genome (*y*-axis). Dot-colors represent the rate of synonymous mutation (*K*s) within each pair of CDS. The central diagonal represents syntenic paralogs between both species, while other chains of dots represent syntenic out-paralogs originating from an ancient whole genome duplication event found throughout the *Oryza* genus. Only CDS pairs with a *K*s ≤ 100 are shown

To analyze the large-scale structure of our *O. longistaminata* genome assembly, we performed a comparative analysis of gene synteny using the *O. sativa* ssp. *japonica* genome as a reference. A genome-wide alignment of all coding sequences (CDS) in each of the two genomes was performed, followed by the identification of syntenic pairs of orthologues (Fig. 2). In total, we identified 9976 pairs of syntenic CDS, which were used to construct the genome-wide alignment. As expected, the genomes of *O. sativa* and *O. longistaminata* appeared highly syntenic, indicated by the central diagonal in Fig. 2. A close-up inspection revealed several minor differences in the CDS order between both genomes, including small-scale (<0.1 Mb) inversions and duplications. In addition to the direct orthologues, also several groups of inter-chromosomal syntenic gene pairs were detected. The median rate of synonymous mutations (*K*s) in those paralogues was 1.37 compared to 0.04 in direct orthologues. This indicated that those inter-chromosomal orthologues are the result of an ancient whole genome duplication event that is conserved in the whole *Oryza* genus^26^. In the *O. sativa* genome, 24 pairs of duplicated segments were found^27^. In our cross-genome alignment, this known pattern of duplications was very well replicated and all major duplication blocks could be detected (Supplementary Figure 3). In summary, genome-wide alignments confirmed that our assembly shows the expected syntenic gene order in the *Oryza* genus, including difficult-to-assemble regions that originated from an ancient genome duplication event.

### Haplotype variations

Since *O. longistaminata* is an outcrossing species, it is reasonable to expect higher haplotype diversity in the *O. longistaminata* genome as compared to the highly inbred cultivated rice species *O. sativa* ssp. *japonica* and *O. sativa* ssp. *indica*. However, this is difficult to exactly quantify, as comparable haplotype-based assemblies are missing. We first focused on SNP-based analyses and aligned the haplotigs back to the final 12 chromosomal sequences using NUCMER^28^ and called SNPs using NUCMER’s show-snp tool. We found that a total of 973,487 bp were different based on the alignments generated by NUCMER. We then analyzed the frequency of small variations (≤10 bp) in different genomic features for bins of 1 Mb (Supplementary Figure 4a). The lowest frequency for such variations was found in the CDS regions with a median of 1.1 variations per kb. The highest frequency was found in the regions 1 kb upstream of each locus with a median of 3.1 variations per kb. This indicates that haplotype diversity is most pronounced in the promoter regions of protein-coding genes, which might have an effect on haplotype-specific promoter activities. To analyze larger variations (>10 bp) we used the output of NUCMER together with Assemblytics^29^. We detected a total of 18,361 larger variations with this approach. Among those were 5,743 deletions, 6,563 insertions, 3,148 repeat contractions, 2,828 repeat expansions, 13 tandem contractions, and 66 tandem expansions. The median occurrence of those variations in bins of 1 Mb was highest in the repetitive content (0.24 variants per kb) but considerably lower in the protein-coding regions with 0.018 variant per kb in the protein-coding loci (Supplementary Figure 4b). In summary, we detected a considerable feature-specific amount of variation between the two parental genomes of *O. longistaminata*. This highlights the complexity of a genome from an outcrossing plant species which is still largely unexplored.

### Repeat content

**Fig. 3:**
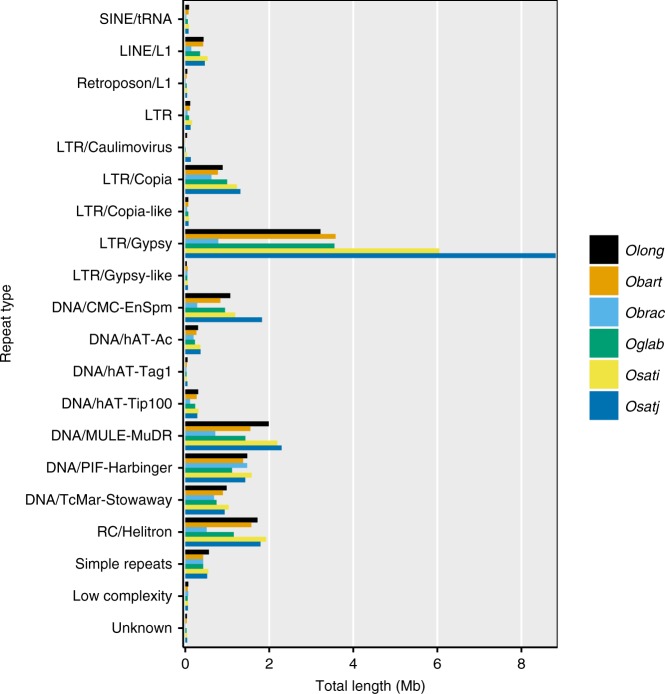
Comparison of repeat-element size in *O. longistaminata* and five related rice species. The total length of selected types of repeat elements is shown. Repeat elements were identified by RepeatMasker. The total size in bp was calculated for each type of repeat element and the top 20 repeat element types in *O. longistaminata* were selected for display. Each color represents one species (*Obart Oryza barthii*, *Obrac Oryza brachyantha*, *Oglab Oryza glabberima, Osati Oryza sativa* ssp. *indica*, *Osatj Oryza sativa* ssp. *japonica*)

Since mobile genomic elements (transposons) are known drivers of genome evolution, we analyzed the transposon content of the *O. longistaminata* genome. Using RepeatMasker^30^ and a database of rice repetitive elements, we found that 38.10% of the total genome assembly consisted of interspersed repeats (Table 2). Further classification of repeat elements revealed that 13.46% of the assembly was classified as long-terminal repeats (LTR) elements and 16.83% was classified as DNA transposons. To put results for *O. longistaminata* into context within the *Oryza* genus, we also analyzed five other rice genomes using the same procedure we used for *O. longistaminata*. The overall amount of repeats in the *O. longistaminata* genome was comparable to the other analyzed rice genome and most similar to the wild African rice species *Oryza barthii*. To further analyze the differences in the observed repeat sizes, especially between the two more repetitive *O. sativa* cultivars and *O. longistaminata*, we plotted the total size of the top 20 most prevalent (by size) repeat elements in *O. longistaminata* for all analyzed species (Fig. 3). LTR retrotransposons from the *Gypsy* family showed the largest absolute changes in size among the analyzed species and are the major contributor to rice genome size differences. In the *O. longistaminata* genome, the total size of *Gypsy* family transposons was most comparable to *O. barthii* and *O. glabberima*. The distribution of repeat elements along the chromosomes followed a repeat-family specific pattern. Repeat elements belonging to the *Copia*, *EnSpm*, and *Gypsy*-family showed the highest density in centromeric regions, while repeat elements of the *Explorer*, *Gaijin*, *Harbinger*, and SINE-type were typically found at lower density in those regions. (Supplementary Figure 5).

**Table 2:** Repeat content in the genome of *O. longistaminata* and five selected rice species.

Species	Genome size (Mb)	Total repeat size (Mb) [% of total sequence]	Retroelements (Mb) [% of total sequence]	DNA transposons (Mb) [% of total sequence]
*Oryza longistaminata*	350.6	133.9 [38.10%]	53.1 [15.11%]	59.1 [16.83%]
*Oryza barthii*	308.3	110.3 [36.08%]	49.4 [16.15%]	48 [15.68%]
*Oryza brachyantha*	260.8	53.3 [21.92%]	18 [7.38%]	31.8 [13.08%]
*Oryza glabberima*	316.4	117.2 [38.65%]	58 [19.13%]	47.7 [15.71%]
*Oryza sativa* ssp. *indica*	427.0	184.5 [44.91%]	106.9 [26.03%]	61.9 [15.08%]
*Oryza sativa* ssp. *japonica*	373.2	171.1 [45.87%]	93 [24.93%]	63 [16.88%]

The *O. longistaminata* genome and five other selected rice species were analyzed by RepeatMasker and values shown were taken directly from the .tbl file. The *O*. *longistaminata* genome used here included the unmapped contigs

### Using the *O. longistaminata* genome in quantitative trait locus (QTL) studies

In our recent work, we applied genotyping-by-sequencing to a population of F2 plants from a cross of *O. longistaminata* and *O. sativa* ssp. *japonica* cv. Nipponbare^23^. In that work, we used the *O. sativa* genome (IRGSP V1.0) as the reference in the initial read mapping step, as no high-quality *O. longistaminata* genome was available at the time. To test the usefulness of our *O. longistaminata* assembly for breeding and mapping applications, we repeated genotyping-by-sequencing and quantitative trait locus (QTL) mapping using the newly assembled *O. longistaminata* genome as the reference.

**Fig. 4:**
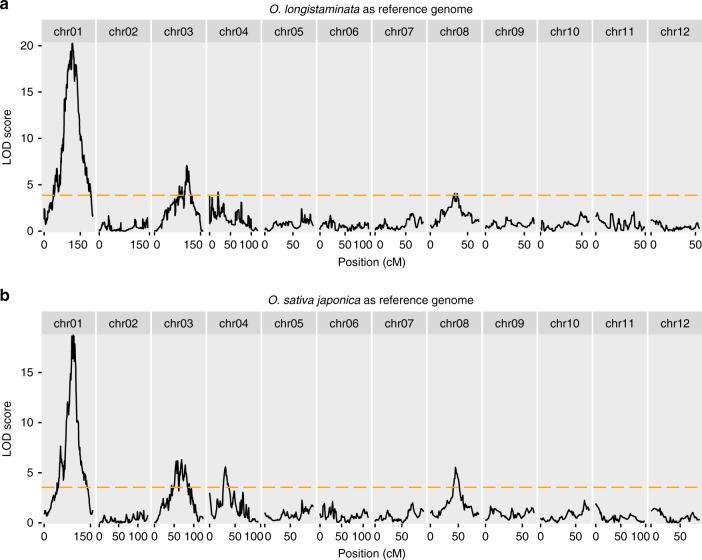
Comparative QTL analysis using two reference genomes. A population of 1081 F2 plants from a cross of *O. longistaminata* and *Oryza sativa* ssp. *japonica* cv. Nipponbare (*O. sativa*) was genotyped. In **a**, the *O. longistaminata* reference genome was used and 2357 SNP markers were detected. In **b**, the *O. sativa* reference genome and 2435 SNP markers were detected. QTL analysis for the number of tiller was performed separately for each of the two reference genomes. The black lines show the LOD scores for the presence of a QTL controlling tiller number using a linear regression model with multiple imputations. The dashed orange lines show the significance threshold for *P* ≤ 0.05 using 100 permutation tests

We used a population of 1081 F2 individuals and set the threshold for missing data per SNP marker to ≤5%. This resulted in 2357 available SNP markers when using the *O. longistaminata* genome as a reference compared to 2435 SNP markers for the NB genome. We proceeded to detect QTL affecting the number of tillers per plant separately for each of the two reference genomes. Using either reference genome, we detected four QTL on chromosomes 1, 3, 4 and 8 (Fig. 4). In addition, the LOD profiles were found to be very similar when the two reference genomes were compared. Based on those results, we concluded that our genome assembly is suitable to be used as a resource in breeding programs involving *O. longistaminata* as a parent.

### Completeness of gene families and metabolic pathways

One major motivation to create a high-quality reference genome is to accelerate functional genomics studies. This requires complete representation of evolutionary (gene families) or functionally (metabolic pathways) defined groups of genes. To this end, we assessed the completeness and the quality of annotations of the set of enzymes that synthesize the phytohormone gibberellic acid (GA) and the SWEET (SUGARS WILL EVENTUALLY BE EXPORTED) family of sugar transport proteins. Using BLASTP and BLASTN searches with *O. sativa* sequences as the query, we detected almost all analyzed genes in the expected chromosomal regions in the *O. longistaminata* genome with a clear 1-to-1 relationship between *O. sativa* and *O. longistaminata* (Supplementary Dataset 1). In general, *O. longistaminata* proteins were very similar (>95% AA identity) compared to *O. sativa* proteins. In five cases (CPS, SWEET1a, 2a, 4 and 11), two highly similar loci are present in the *O. longistaminata* genome, as opposed to a single locus in *O. sativa*. This pairs of loci were always found in close proximity to each other. It is conceivable that, e.g., highly heterozygous parts of the genome could lead to breaks in the assembly and the observed duplication in reality represents two regional haplotypes that were not correctly picked up during haplotype phasing.

### Identification of functionally enriched genomic regions

**Fig. 5:**
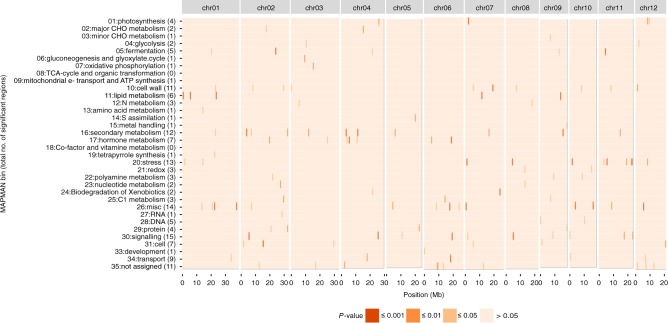
Functional enrichment in genomic regions. The *O. longistaminata* genome was divided into 654 regions of 0.5 Mb each. Within each region, the number of genes associated with MAPMAN functional categories shown on the left was determined. Significant enrichment of functional categories was tested by Fisher’s exact test and *P*-values were corrected by the Bonferroni–Holm method. *P*-values for each genomic region (*x*-axis) and functional category (*y*-axis) are color-coded according to common significance thresholds as indicated in the legend

Recently, it was reported that barley chromosomes feature genomic compartments, which are characterized, among other features, by an enrichment in specific gene functions^18^. We could not detect such clear compartments in our *O. longistaminata* genome assembly, most likely because rice genomes are approximately one order of magnitude smaller compared to barley (5 Gb vs. <0.5 Gb) and are thus less compartmentalized. However, by analyzing the distribution of MAPMAN functional gene categories along the chromosomes, we discovered 153 genomic regions (0.5 Mb) in which at least one functional category of genes was significantly (*P* ≤ 0.05) enriched (Fig. 5). The functional categories which were enriched most often were 30:signaling (15 regions), 26:misc (14 regions), 20:stress (13 regions), and 16:secondary metabolism (12 regions). Multi-locus arrangements of very similar genes are often collapsed in assemblies based on short reads, which is highly undesirable as several agriculturally important alleles conferring resistance to biotic stresses are part of large, multi-gene clusters^31–33^. The 13 genomic regions, in which stress-related genes were significantly enriched contained between 7 and 20 stress-related genes each. Evaluating the potential of those regions for biotic stress resistance, e.g., by a targeted breeding approach, might be one way to utilize this reference genome assembly.

### Gene expression profiles

**Fig. 6:**
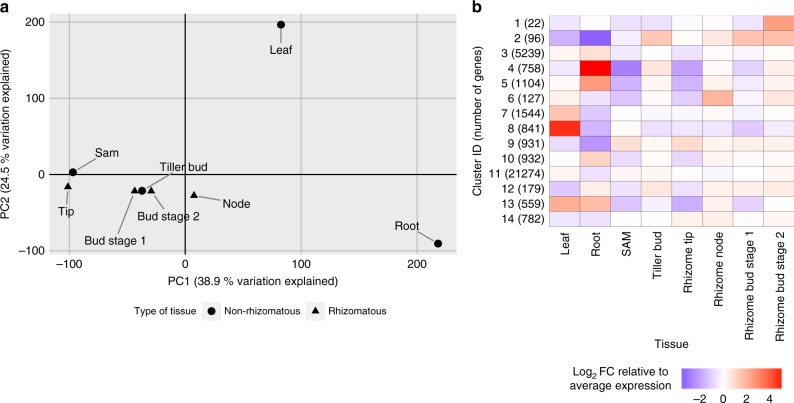
Analysis of gene expression in eight *O. longistaminata* tissues. **a** A principal component analysis using scaled and centered RPKM data from the eight indicated tissues was performed. For each tissue, the scores from the first two principal components (PC) were plotted along with the percentage of the explained variation. Symbols indicate non-rhizomatous and rhizomatous tissues, respectively. **b** A heatmap representation of gene expression in the eight indicated tissues is shown. Each row represents one cluster of genes and the average expression within each cluster is shown. Before averaging, gene expression was normalized by transforming RPKM values to the log_2_-fold change relative to the average gene-wise expression. Clustering was performed using the *k*-means method and *k* = 14 was chosen to minimize both, the number of clusters and the within-cluster sum-of-squares (elbow method)

RNAseq data from eight diverse tissues (leaf, root, shoot apical meristem, tiller bud, rhizome tip, rhizome node, rhizome bud stage 1 and 2) was used to detect genes in the *O. longistaminata* genome in combination with computational gene prediction (Supplementary Figure 6 and Supplementary Table 2). In total, 34,389 loci were detected and their expression was quantified (Supplementary Dataset 2). A principal component analysis of gene expression data indicated similar expression profiles in related rhizomatous and non-rhizomatous tissues (Fig. 6a). Gene expression in rhizome tips was found to be most similar to the shoot apical meristem of above ground shoots, while samples from rhizome buds and tiller buds were most similar with respect to each other. To detect patterns of gene expression and tissue-specific genes, *k*-means based clustering was performed with *k* = 14 (Fig. 6b). We found 841 genes in cluster 8 expressed primarily in the leaf. Genes involved in photosynthesis (light reaction, photorespiration, Calvin cycle) and secondary metabolism (flavonoids, phenylpropanoids, isoprenoids) were significantly enriched (*P* < 0.05) in that cluster based on MAPMAN functional annotations (Supplementary Dataset 3). Similarly, 758 genes in cluster 4 were primarily expressed in the roots and genes putatively encoding peroxidases and glutathione-S-transferases were enriched among those genes. The clusters 1 (22 genes), 2 (96 genes), and 6 (127 genes) contained genes which were specifically expressed in a combination of rhizome and meristematic (shoot apical meristem, tiller bud) tissues.

## Conclusion

In this work, we presented a high quality, haplotype-aware reference genome for the wild rice species *O. longistaminata*. We challenged our assemblies’ usefulness for QTL mapping and functional genomics, and concluded that this work provides a solid basis for future efforts to understand and transfer useful traits from *O. longistaminata* into cultivated rice species. In comparison to other chromosome-level assemblies^3,4^, our approach is based on assembling whole-genome shotgun sequencing data directly followed by exploiting gene synteny and a genetic map to form pseudo-chromosomal sequences. This greatly simplified the sequencing and post-assembly workflow. Our work provides a glimpse into the possibility for semi-automated genome assemblies for genera of crop species, in which at least one high-quality reference genome is available. This would allow pan-genomic approaches for selected genera^34^.

## Methods

### Plant material

*O. longistaminata* (accession IRGC110404) was used for whole genome and transcriptome sequencing. Plants were cultivated in a controlled environment chamber at Nagoya University in Nagoya, Aichi Prefecture, Japan. The F2 populations used for QTL analysis were described previously^23^.

### DNA and RNA sequencing

For long read-sequencing, DNA was extracted by the ISOPLANT method^35^. In total, 20 µg of DNA (106 ng µL^−1^) was used for sequencing on a Pacific Biosystems RS instrument using P6v2 chemistry and 16 SMRT V3 cells (Supplementary Figure 1). Sequencing was carried out by Macrogen (Seoul, South Korea). For short read-sequencing, DNA from the same individual was extracted using the same method. Libraries were prepared using the TruSeq DNA v2 kit and sequenced on an Illumina Genome Analyzer IIx platform (Illumina, San Diego, CA, USA). For transcriptome sequencing, RNA was extracted from leaves, roots, tiller buds, shoot apical meristems, and rhizome tissues including early (stage 1) and late (stage 2) buds, tips, and node regions of mature rhizomes using the QIAGEN plant RNA kit (Hilden, Germany) (Supplementary Figure 5). Paired-end reads were generated on a HiSeq2000 platform.

### Genome sequence assembly

Raw SMRT sub-reads were first assembled using the FALCON assembler (https://github.com/PacificBiosciences/FALCON-integrate, release v2.1.2) to generate primary contigs. FALCON was configured as follows: genome_size = 350000000, length_cutoff = 5000, length_cutoff_pr = 5000, pa_DBsplit_option = -x500 -s200, pa_HPCdaligner_option = -v -B4 -e.70 -l1000 -s1000, falcon_sense_option = --output_multi --min_idt 0.70 --min_cov 4 --max_n_read 400, ovlp_DBsplit_option = -x500 -s200, ovlp_HPCdaligner_option = -v -B4 -h60 -e.96 -l500 -s1000, overlap_filtering_setting = --max_diff 50 --max_cov 50 --min_cov 3 --bestn 10. The primary assembly was then haplotype-phased using FALCON-UNZIP (obtained at: https://downloads.pacbcloud.com/public/falcon/falcon-2018.03.12-04.00-py2.7-ucs2.tar.gz) and default settings.

After assembly and haplotype phasing, error correction was performed in two steps. First, all SMRT reads were realigned to the assembly using quiver integrated in SMRT analysis (v.2.3.0). Quiver polishing was performed by filtering of subreads (minLength = 50, minSubReadLength = 50, readScore = 0.75), mapping of subreads to the assembly using blasr (maxHits = 10, maxDivergence = 30, minAnchorSize = 12, seed = 1, minAccuracy = 0.75, minLength = 50, algorithmOptions = -useQuality) followed by error correction. The resulting sequences were again polished by first aligning paired-end short reads using bwa-mem^36^ followed by error-correction using Pilon^37^ with the --diploid, --nostrays, and --fix indels options. The same procedure was performed for primary contigs and associated haplotype sequences (haplotigs).

The polished contigs were then arranged in a linear fashion using ALLMAPS^38^ with two different genome maps. The first map consisted of 301 high-confidence SNP markers^23^. To establish their respective positions in the polished contigs, a 200 bp genomic DNA sequence from *O. sativa* ssp. *japonica* cv. Nipponbare surrounding the SNP was mapped to the polished contigs using bwa-mem. The alignment was converted to bed format using bamtobed from bedtools v2.25.0^39^ and converted to an ALLMAPS map by a custom R script. The second map exploited gene synteny between *O. longistaminata* and *O. sativa*. Sequences representing all gene models from IRGSP1.0 reference genome build were obtained from http://rapdb.dna.affrc.go.jp/ and aligned to the polished contigs using BLASTN. The best blast hit from the first isoform for each gene model was isolated and used to create a gene synteny-based map. Spurious blast hits (blast hits from a specific *O. sativa* chromosome with a run-length = 1) from contigs with three or more hits were removed. Only the start coordinates for each gene model were used, except for contigs with only 1 blast hit. In that case also the stop coordinate of the gene model was used to allow orientation. In total, this map contained 34,097 anchor points. The combined genetic maps were first used to detect putative chimeric contigs. Using the jcvi.assembly.allmaps split command with the –chunk = 4 option 37 breakpoints were identified and the contigs were split accordingly. Both maps were remade to represent the split contigs and were finally used as input for the jcvi.assembly.allmaps path command. The resulting 12 pseudo-chromosomes together with all unmapped contigs (7.8% of the total genome, merged into one DNA sequence with 1 kb separating the contigs) is referred to as the *O. longistaminata* reference genome V2.0 (respecting the V1.0 assembly described earlier^5^). Completeness of the assembly was assessed using BUSCO 2.0.1 with the embryophyta_odb9 dataset^24^.

### Genotyping and QTL detection

Genotyping based on SNPs and subsequent QTL detection were performed as described in detail in our previous work^23^. In short, 1081 F2 plants of a cross of *O. longistaminata* and *O. sativa* ssp. *japonica* cv. Nipponbare were used for genotyping-by-sequencing using the TASSEL4 pipeline^40^. SNPs were filtered based on minor allele frequency, parental alleles, read depth, and missing data. As the last step, putative errors were corrected and missing data was imputed based on flanking alleles. Phenotyping was performed by digging up plants from the paddy field and counting all shoots for each individual plant. In addition, six replicate plants of each of the two parents were used for genotyping and phenotyping. QTL detection was performed using the R/qtl package^41^. A linear regression model with multiple imputations implemented in the scanone function was used. The threshold for significance was calculated from 100 permutation tests.

### Genomic feature detection

The final *O. longistaminata* reference genome V2.0 was annotated using a combination of in silico gene prediction and transcriptome data. Gene prediction was carried out using the MEGANTE gene prediction pipeline^42^ using the profile for *O. sativa* and standard settings. In addition, RNAseq data from eight different tissues described above was used to identify expressed genes. For this, raw RNAseq reads were first cleaned using Trimmomatic-0.36^43^ with the options LEADING:3, TRAILING:3, and MINLEN:30 followed by re-pairing reads using pairfq (https://github.com/sestaton/Pairfq). Cleaned reads were then aligned separately for each tissue to the *O. longistaminata* reference genome V2.0 using hisat2^44^ with the –dta option. Transcripts were assembled from read alignments using stringtie^45^ with the –m 50 option and all eight resulting GTF-files were merged using stringtie with the –merge argument. TransDecoder (https://github.com/TransDecoder/TransDecoder) was used to derive transcript sequences and detect all ORFs longer than 50 AA (TransDecoder.LongOrfs -m 50). The likely coding region for each transcript was detected using TransDecoder.Predict supplemented by BLASTP data obtained by querying the SWISS-PROT database with the longest ORF of each transcript. Predicted and experimentally verified gene models were combined using a custom R/Bioconductor script and in case of overlaps precedence was given for the experimentally verified gene models. Repeat elements in the final *O. longistaminata* assembly were identified using RepeatMasker with the options: -pa 4 -x -excln -html -gff -no_is -species rice. Putative centromeric regions were identified using a 154 bp monomer isolated from the *O. sativa* CentO region (AY101510.1) as a query for BLASTN searches against the *O. longistaminata* genome.

### Functional gene annotations

Functional annotations for MEGANTE-predicted genes including BLASTP hits, cDNA accessions numbers from similar cDNAs, Interpro domains, and GO terms were used as reported by MEGANTE. Expressed genes were annotated using the best BLASTP hit using a protein database containing all AA sequences from UniProt with taxon ID 4527 (*Oryza* genus). Based on the UniProt accession number, Interpro domain IDs and GO terms were added. A short human-readable description was added by using the name of the most specific ortholog group derived from eggnog 4.5.1^46^. In addition, all final gene models (using the protein encoded by the first reported splice variant) were assigned to a MAPMAN functional annotation using Mercator^25^.

### Genome-wide alignments

Analyses of genome synteny were performed using the CoGe platform (https://genomevolution.org/coge/)^47^. Whole genome sequences and all CDS as GFF annotation from the *O. longistaminata* reference genome and from the *O. sativa* genome were uploaded to CoGe. CDS from both genomes were aligned using LastZ (--hspthresh 3000). DAGChainer (using relative gene order, -D 20 and -A 5) was used to identify chains of syntenic genes and the Quota Align option was used to merge neighboring syntenic regions. The results file including synonymous mutation rates was downloaded and parsed using a custom R script before plotting.

### Haplotype analysis

For analysis of the error-corrected haploid-phased alternative contigs (haplotigs), each sequence was aligned to the final 12 chromosomes of the *O. longistaminata* reference genome using NUCMER version 3.1^28^ with the options: –maxmatch –l 100 –c 500. For small variations, the show-snps program from NUCMER was used with the options –Clr –x 1 –T and the resulting table was converted to a vcf file using the MUMmerSNPs2VCF.py program found here: (https://github.com/liangjiaoxue/PythonNGSTools/blob/master/MUMmerSNPs2VCF.py). The resulting vcf file was parsed into R and only variations with a length of ≤10 bp in both the REF and ALT fields were used for further analysis. The output of NUCMER was also analyzed using Assemblytics^29^ and the resulting BED-file was parsed into R and only variations with a length of >10 bp in either the ref_gap_size or the query_gap_size field were used for further analysis.

### General data processing

Principal data analysis and visualization was performed using R V3.3.2 or later. Quantification of gene expression was performed using Rsubread^48^. *K*-means based clustering was performed using MBCluster.Seq^49^. Principal component analysis was performed using the prcomp function. Enrichment of functional categories in genomic regions or expression-based clusters was tested for by Fisher’s exact test followed by Bonferroni–Holm correction using the fisher.test and p.adjust functions, respectively. Manipulation of biological sequences and genome-based features was performed using Bioconductor V3.3 and the packages Biostrings, rtracklayer, and GenomicRanges. General data visualization was performed using ggplot2. Circular plots of genome features were created using Circos^50^.

## Electronic supplementary material

Supplementary Material

Description of additional supplementary items

Dataset 1

Dataset 2

Dataset 3

## Data Availability

All reads used for genome (Bioproject PRJDB6339) and transcriptome (Bioproject PRJDB6351) assembly have been uploaded to the DNA Databank of Japan (DDBJ). A genome browser and other *O. longistaminata* genome-related data including all sequences and annotations can be found at http://olinfres.nig.ac.jp/
