# Supplementary material for: Assembling the genome of the African wild rice Oryza longistaminata by exploiting synteny in closely related Oryza species
Source: Commun Biol. 2018 Oct 5;1:162. doi: 10.1038/s42003-018-0171-y (PMC6173730; doi:10.1038/s42003-018-0171-y)
Supplement: Supplementary file 1 — Supplementary Material [file 42003_2018_171_MOESM1_ESM.pdf]

# Supplementary Figures

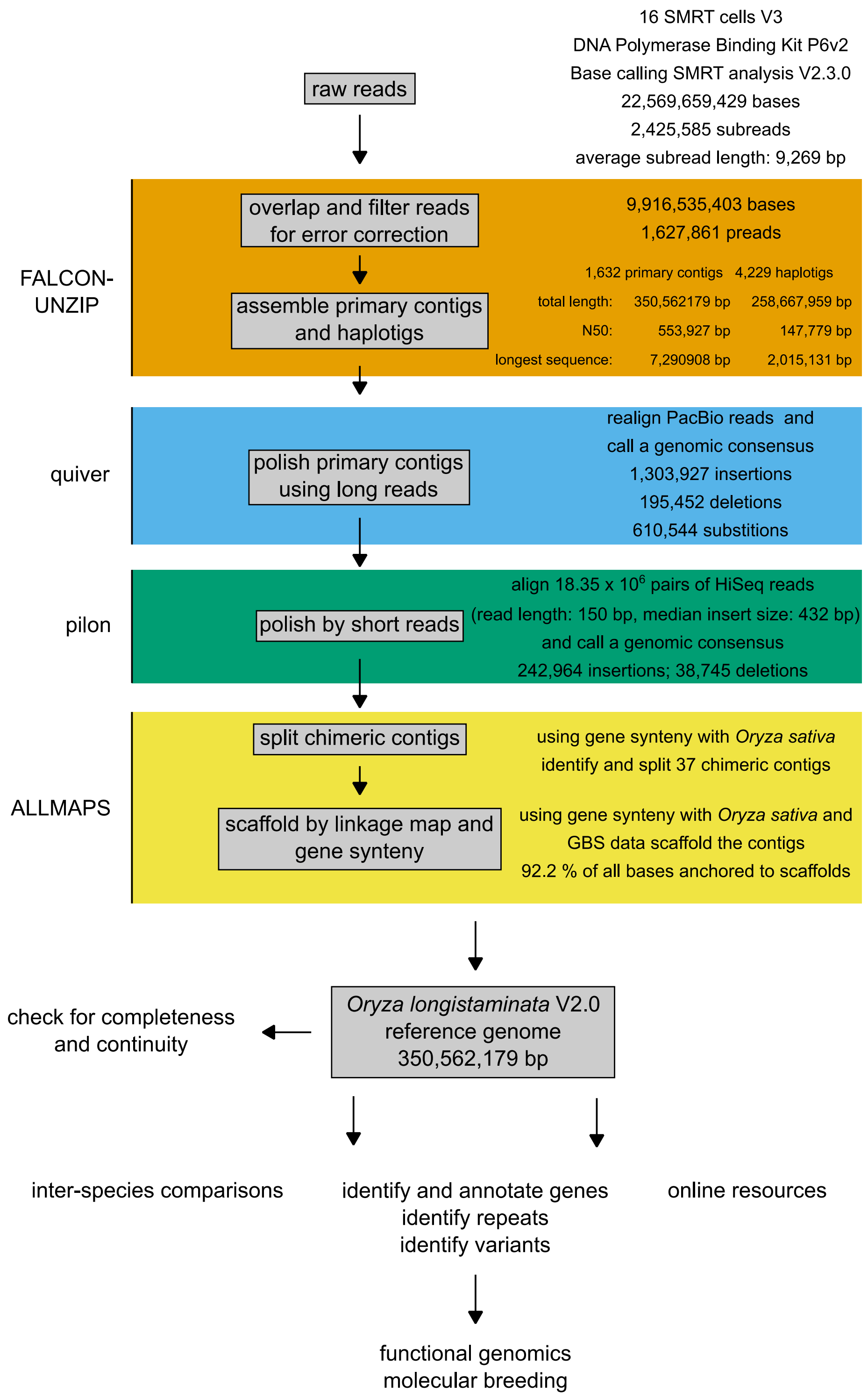


## Supplementary Figure 1: Genome sequencing and assembly strategy.

A flowchart showing the key steps in sequencing, assembling, error-correcting and scaffolding the *O. longistaminata* genome. Principal software used at each stage is shown on the left.

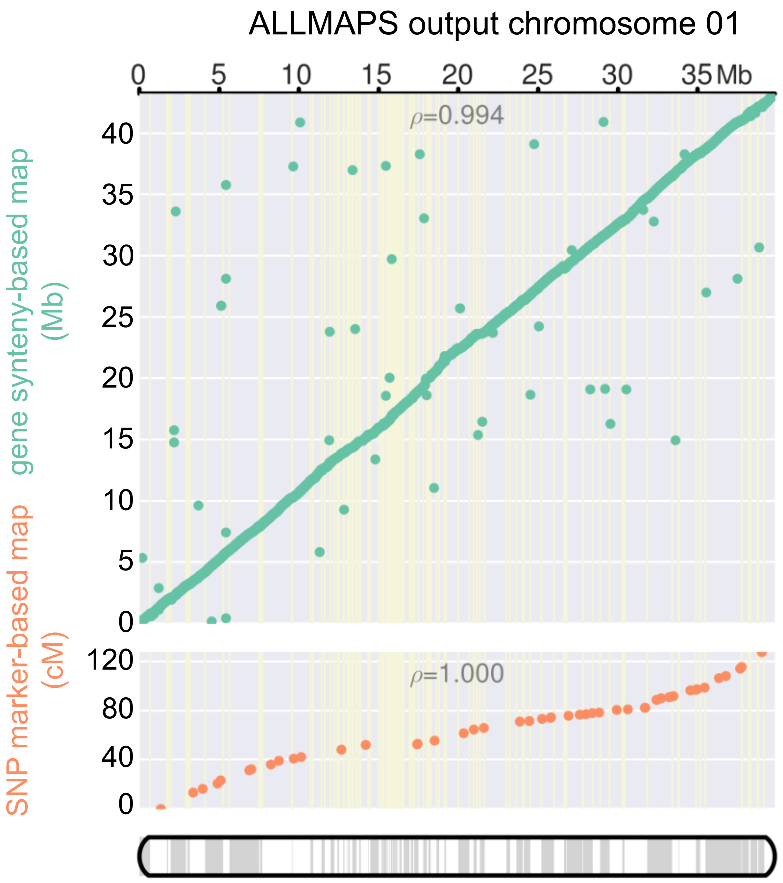


Supplementary Figure 2: Scaffolding of *O. longistaminata* chromosome 1 using two genome maps.

Scaffolding the *O. longistaminata* genome was performed using ALLMAPS. As an example, a modified overview figure created by ALLMAPS for chr01 is shown. The top axis represents the final *O. longistaminata* chromosome 1. The left axes represent the positions of anchors on each of the two genome map. Each dot represent one anchor. In the upper scatterplot each dot represent one gene (position in bp). In the lower scatterplot each dot represent one SNP marker (position in cM). The ρ-value on each scatter plot measures Pearson's correlation coefficient, with 1 indicating perfect co-linearity.

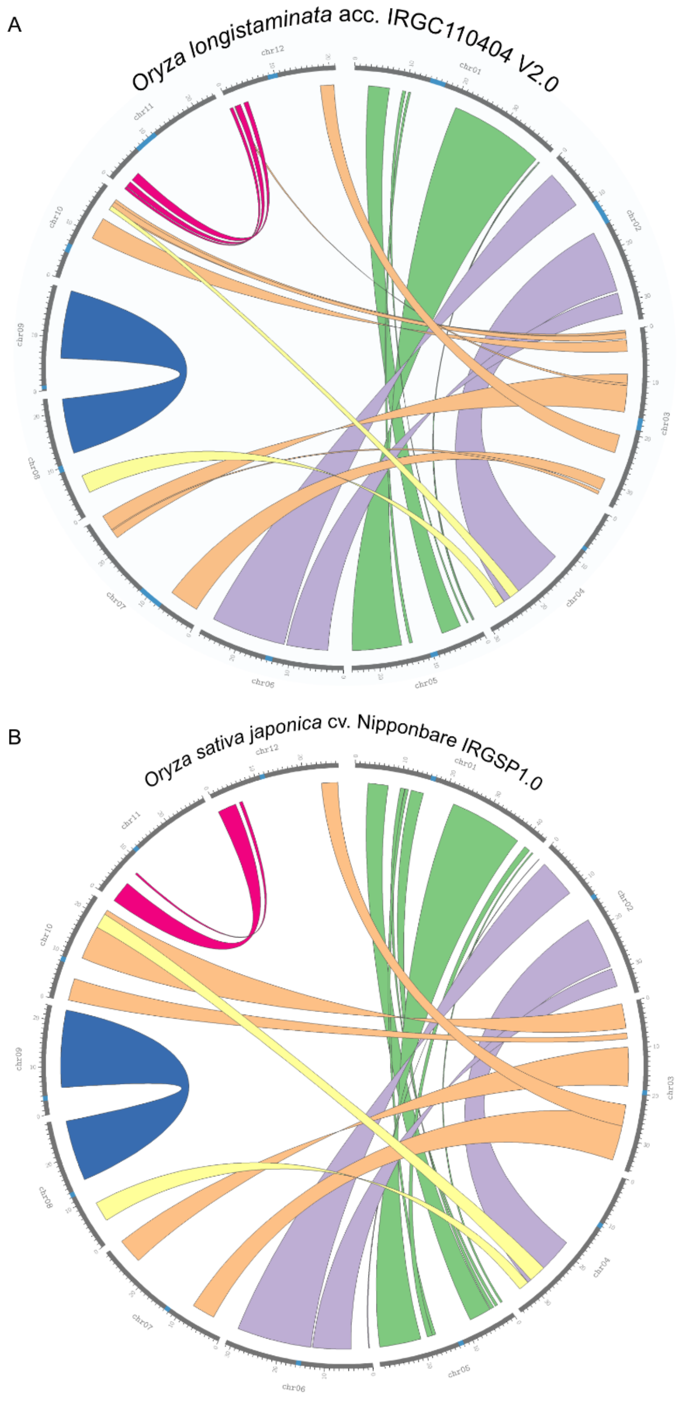


## Supplementary Figure 3: Segmental duplications originating from an ancient whole-genome duplication event.

Segmental duplications in the *O. longistaminata* genome (**A**) and the *Oryza sativa japonica* genome (**B**) were analyzed separately by using within species genome-wide alignments of CDS. Data was generated in the same way as described for the cross-species alignment of *O. longistaminata* and *O. sativa japonica*. Grey bars represent the chromosome backbones and blue parts show the centromeric region. Coordinates are given in Mb. Links between chromosomes show regions with highly syntenic chains of CDS. Colors represent links originating from chromosomes 1, 2, 3, 4, 8 and 11, respectively.

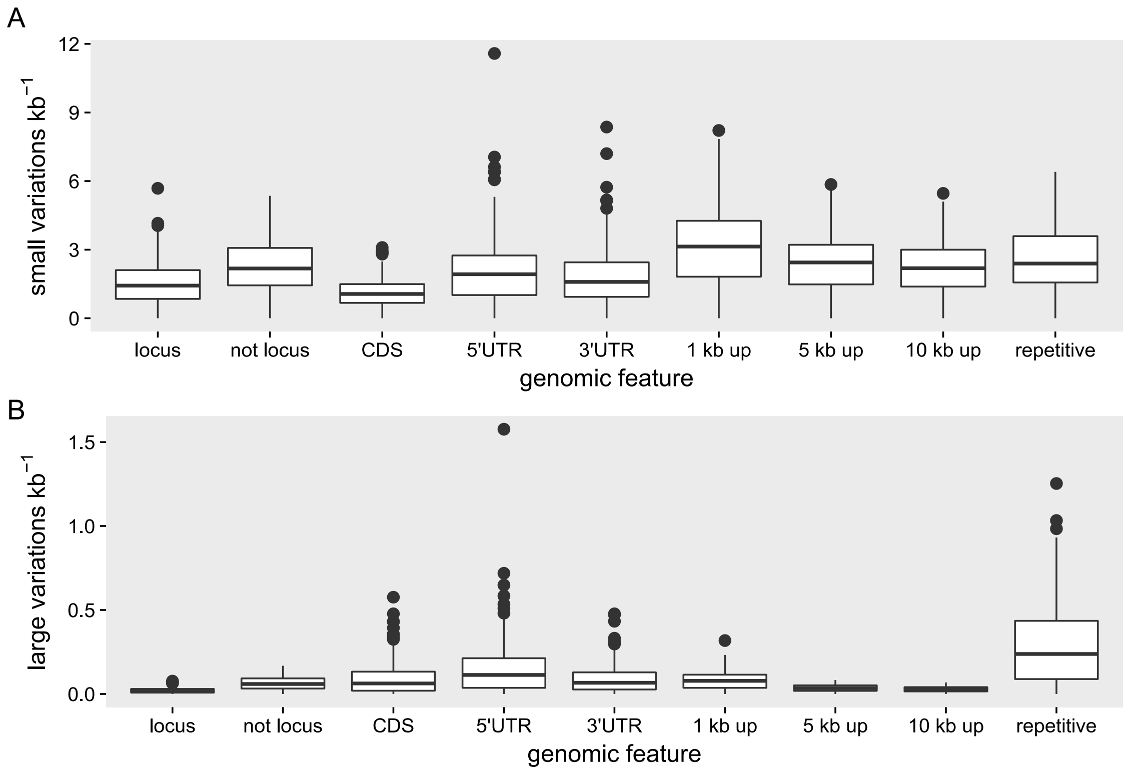


## Supplementary Figure 4: Occurrence of small and large inter-haplotype variations.

Small (**A**) (≤ 10 bp) and large (**B**) (> 10 bp) variations were analyzed in the *O. longistaminata* genome and summary statistics were calculated for bins of 1 Mb and displayed as occurrences per 1 kb of the indicated feature. Variations in (**A**) included SNPs and small indels called directly from a NUCMER alignment, while (**B**) shows the output of Assemblytics including larger indels and changes in repeat size. "Locus" indicates whole protein-coding loci (including UTRs), "not locus" is the inverse selection of the previous. "CDS" indicates only protein-coding sequences. "5'UTR" and "3'UTR" indicate non-coding 5' and 3' regions. "1 kb up", "3 kb up" and "10 kb up" indicate regions of the respective lengths upstream of genomic loci containing putative promoter elements. "repetitive" indicates all repeat elements identified by repeat masker as described earlier.

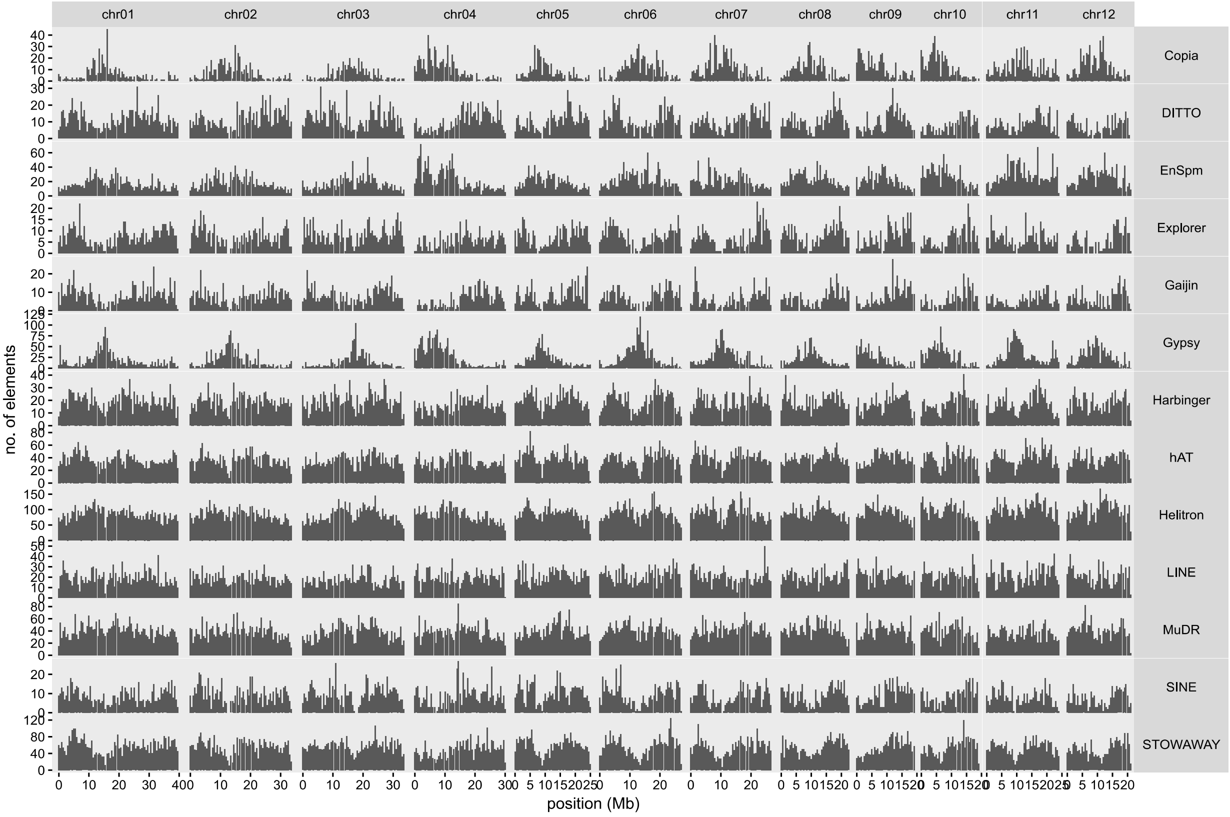


Supplementary Figure 5: Distribution of repeat elements along the *O. longistaminata* chromosomes.

The *O. longistaminata* genome was divided into 654 regions of 0.5 Mb each. The number of repeat elements in each region is plotted separately for common families of repeats.

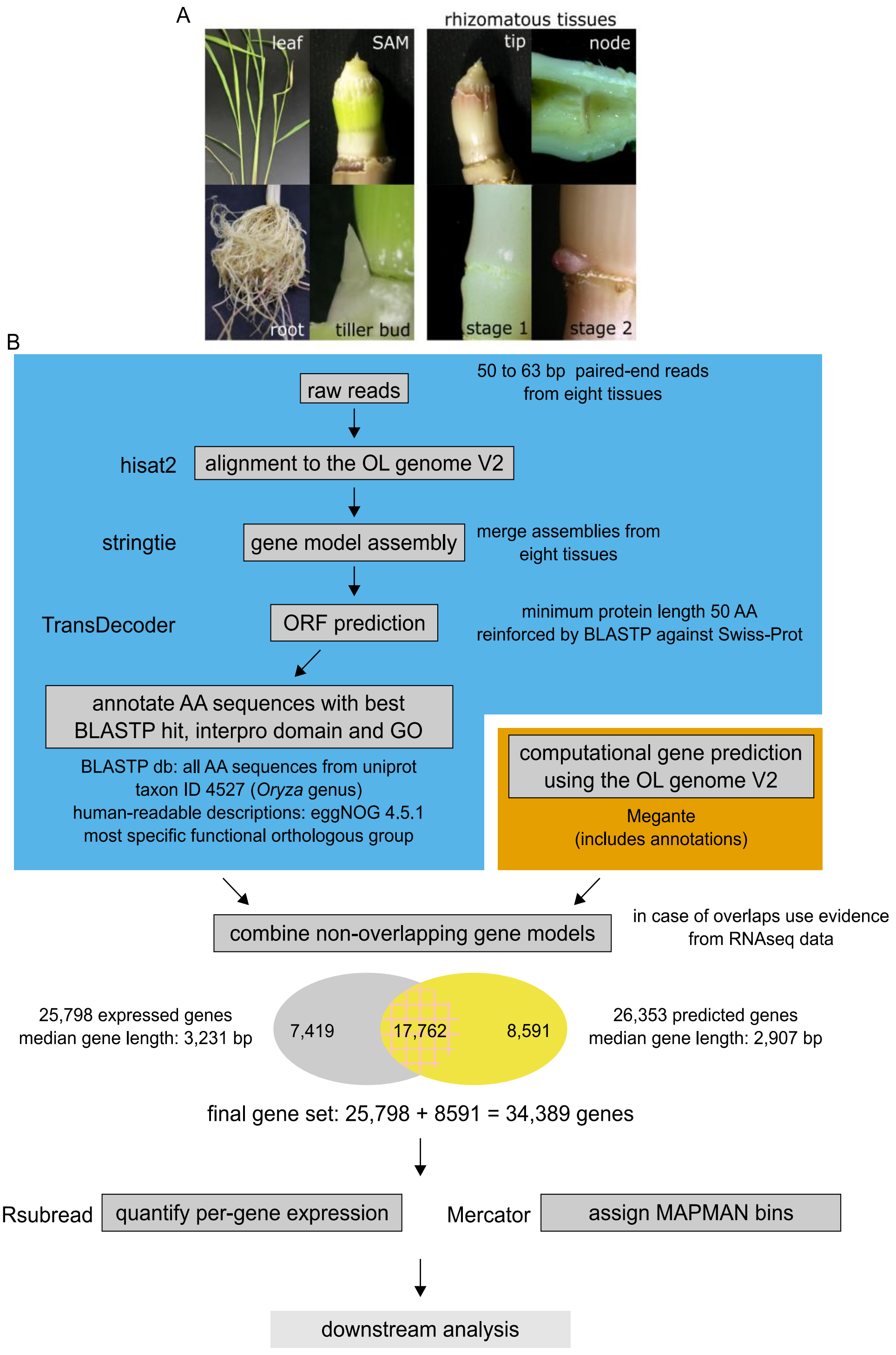


## Supplementary Figure 6: Transcriptome assembly strategy.

The construction of the *O. longistaminata* reference transcriptome is described. (**A**) The eight analyzed tissues are shown. (**B**) In the upper part a flowchart describing the assembly and annotation of a set of expressed genes is shown (blue box). In the lower part the merging of gene models based on expression data and computational gene prediction is described.

# Supplementary Tables

## Supplementary Table 1: Performance of the Canu assembler compared to FALCON and FALCON UNZIP.

Seven different assemblies using the Canu assembler are compared to the final primary assembly from FALCON-UNZIP. FALCON v3 indicates a previous assembly with FALCON without haplotype phasing. For each Canu assembly the read correction and trimming steps were carried out with standard parameters and then parameters in the final assembly step were varied. Canu (default) indicates Canu's default settings for cer (allowed corrected error rate) and mol (minimum overlap length).

| **assembler** | **cer** | **mol** | **contigs** | **total length (bp)** | **N50 (bp)** | **longest contig (bp)** |
|---|---|---|---|---|---|---|
| FALCON-UNZIP | - | - | 1,632 | 350,62,179 | 553,927 | 7,290,908 |
| FALCON v3 | - | - | 2,889 | 339,530,447 | 260,911 | 1,907,208 |
| Canu (default) | 0.045 | 500 | 10,393 | 584,043,527 | 95,553 | 1,028,605 |
| Canu | 0.09 | 400 | 9,792 | 537,431,782 | 109,464 | 1,404,122 |
| Canu | 0.09 | 300 | 9,801 | 537,603,161 | 109,747 | 1,404,091 |
| Canu | 0.09 | 200 | 9,864 | 537,768,040 | 106,792 | 1,418,225 |
| Canu | 0.18 | 400 | 9,729 | 523,874,801 | 116,051 | 2,160,673 |
| Canu | 0.18 | 300 | 9,750 | 525,297,358 | 116,466 | 2,160851 |
| Canu | 0.18 | 200 | 9,729 | 523,873,801 | 116,051 | 2,160,673 |

## Supplementary Table 2: Mapping statistics of the *O. longistaminata* reference transcriptome.

Mapping statistics of RNAseq reads aligned to the reference genome for transcriptome assembly and quantification are shown. Reads were aligned using the hisat2 aligner. Total reads is the number of forward reads after trimming, pairing ratio is the ratio of forward reads that have a mate and the overall alignment ratio is shown as reported by hisat2.

| tissue | total reads | pairing ratio (%) | overall alignment ratio (%) |
|---:|---|---|---|
| leaf | 19,121,588 | 82.76 | 89.9 |
| rhizome bud stage 1 | 13,315,856 | 81.56 | 93.54 |
| rhizome bud stage 2 | 27,274,491 | 77.79 | 81.11 |
| rhizome node | 10,908,488 | 81.54 | 91.04 |
| rhizome tip | 13,787,425 | 81.37 | 92.3 |
| root | 9,773,000 | 78.12 | 87.96 |
| shoot apical meristem | 23,603,101 | 80.78 | 92.45 |
| tiller bud | 18,909,867 | 82.09 | 93.32 |

# Supplementary Notes

## Supplementary Note 1: Assembly of the *O. longistaminata* genome using an alternative assembler

In addition FALCON-UNZIP we also evaluated the performance of the Canu assembler (v1.6) with our dataset. We first performed an assembly using standard parameters for uncorrected PacBio reads and an estimated genome size of 340 Mb. This resulted in a primary assembly of 10,393 contigs with a total length of 584 Mb and an N50 of 95.6 kb. Compared to the FALCON assembly (350.6 Mb on 1,632 contigs, N50 554 kb) the canu assembly lacks continuity and is about 230 Mb larger than the intended target size.

Closer inspection of the results from Canu's initial read correction and read trimming indicated that those two steps were carried out satisfactory. During those steps our read dataset was reduced from 66-fold coverage to 32-fold coverage, which is in agreement with the recommended outcome of those steps. We then tried to improve the Canu assembly by using the corrected, trimmed read set with varying key parameters in the final unittiging step. To improve continuity we increased the allowed correctedErrorRate to 0.09 and 0.18 (default: 0.045) while also decreasing minOverlapLength to 400, 300 and 200 (default: 500 bp). This resulted in a total of six additional assemblies (Supplementary Table 1). However, using this strategy we could only achieve a marginal improvement of continuity. We therefor concluded that the assembly by FALCON is superior compared to Canu and used this assembly for further analyses. It can be speculated that the higher heterozygosity of *O. longistaminata* (compared to inbred commercial varieties like Nipponbare or Shuhui498) is the reason for the bad performance of Canu. FALCON-UNZIP, on the other hand, seems to be designed to specifically assemble heterozygous or higher-ploidy genomes. This is supported by the fact that in our hands Canu produced highly continuous assemblies when used with raw data from other, more inbreed rice varieties.
