# Supplementary material for: Assembling the genome of the African wild rice Oryza longistaminata by exploiting synteny in closely related Oryza species
Source: Commun Biol. 2018 Oct 5;1:162. doi: 10.1038/s42003-018-0171-y (PMC6173730; doi:10.1038/s42003-018-0171-y)
Supplement: Supplementary file 2 — Description of additional supplementary items [file 42003_2018_171_MOESM2_ESM.docx]

# Description of additional supplementary items

## Supplementary Datasets

(as separate .xlsx files)

## Supplementary Dataset 1: Functional and evolutionary gene family completeness.

Contains detailed information of selected groups of genes and their representation in the new *O. longistaminata* reference genome to assess completeness of the annotated genes.

## Supplementary Dataset 2: Gene expression data from eight *Ol* tissues.

Contains gene expression data from eight diverse tissues and further annotations of all genes.

## Supplementary Dataset 3: Functional enrichment analysis on genes sets determined by expression-based k-means clustering.

Contains information on enriched functional MAPMAN terms in gene sets determined by k-means clustering of expression data from eight diverse tissues.
